# Association pityriasis rubra pilaire et myasthénie

**DOI:** 10.4314/pamj.v10i0.72213

**Published:** 2011-09-21

**Authors:** Fatima Zahra Agharbi, Amal Elbekkal, Hanane Baybay, Mariame Meziane, Ouafae Mikou, Fatima Zahra Mernissi

**Affiliations:** 1 Service de dermatologie CHU HASSAN II, Fès, Maroc

**Keywords:** Pityriasis rubra pilaire, myasthénie, maladies auto-immunes, infections, anti TNF alpha

## Abstract

Le pityriasis rubra pilaire est un trouble de kératinisation rare dont l’étiopathogénie reste inconnue mais dont l'association avec autres pathologies a été déjà rapportée. Nous rapportons l'observation d'une jeune patiente qui présente un pityriasis rubra pilaire associé à une myasthénie. Traitée par Néostigmine et thymectomie avec bonne évolution sur le plan neurologique. Vu son désir de grossesse un traitement systémique de son pityriasis rubra pilaire n'a pas pu être instauré et l’évolution sous dermocorticoïdes n’était pas très favorable.

## Introduction

Le pityriasis rubra pilaire (PRP) est un trouble de kératinisation rare, caractérisé par des papules folliculaires kératosiques qui confluent en plaques avec hyperkératose palmoplantaire jaune orangée. C'est une pathologie dont l’étiopathogénie reste inconnue, cependant nombreux cas d'association PRP et autres maladies notamment auto-immunes ont été rapportés.

## Patient et observation

**Figure 1:**
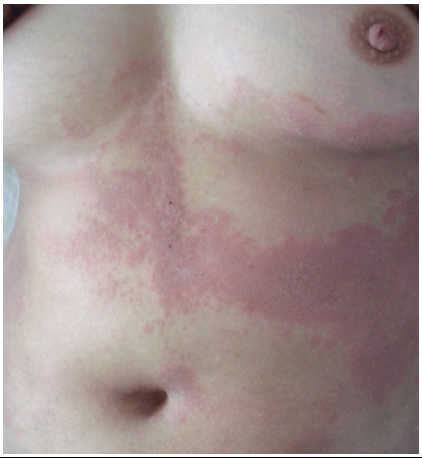
papules érythémateuses folliculaires confluentes en plaques chez une patiente présentant un pityriasis rubra pilaire associé à une myasthénie et prise en charge au CHU Hassan II de Fès, Maroc.

Il s'agit de Mme O.N âgée de 33 ans, nullipare, sans antécédents pathologiques notables. Elle présentait depuis 5 ans une éruption érythémato squameuse non prurigineuse au niveau du tronc, membres supérieurs et inférieurs, associée à une fatiguabilité fluctuante s'aggravant à l'effort, une lenteur de parole, une dysphagie et une chute des paupières. L'examen trouvait de multiples papules érythémateuses folliculaires confluentes par endroits en plaques hérissées de papules folliculaires en périphérie (Figure. 1), une kératodermie palmo-plantaire jaune orangée, un état squameux avec des papules folliculaires érythémateuses du cuir chevelu et un effluvium télogène, dystrophie unguéale diffuse. Le reste de l'examen somatique avait objectivé un déficit musculaire distal des deux membres supérieurs, un ptosis bilatéral avec une dysarthrie. La biopsie cutanée était non spécifique. L’électromyogramme avait objectivé un bloc myasthénique post synaptique. Le test à la prostigmine après 30 minutes et les AC anti récepteurs à l'acétylcholine étaient positifs. Le diagnostic de PRP type I associé à une myasthénie a été retenu sur l'aspect typique des lésions cutanées, les résultats de l’électromyogramme, la réponse à la prostigmine et la positivité du bilan immunologique. La patiente a été mise sous dermocorticoïdes et Néostigmine puis elle a bénéficié d'une thymectomie dont l’étude anatomopathologique était sans particularité. La patiente a refusé tout traitement systémique de son PRP vu son désir de grossesse.

## Discussion

Le pityriasis rubra pilaire (PRP) est un syndrome cutané particulier qui associe des lésions érythémato-squameuses et une kératodermie palmo-plantaire jaune orangée. C'est une affection rare qui touche les 2 sexes à la même fréquence. Deux pics de fréquence sont observés: les dix premières années de vie et entre 40 et 60 ans. Griffiths a décrit 5 types de PRP selon l’âge de survenue, la clinique et le pronostic: type I: PRP classique de l'adulte, type II: la forme atypique de l'adulte, type III: PRP juvénile classique, type IV: PRP juvénile circonscrit, type V: PRP juvénile atypique [1, 2]. Récemment un sixième type de PRP a été proposé c'est le PRP associé au HIV. Le PRP classique de l'adulte est la forme la plus fréquente, elle réalise des papules folliculaires érythémateuses de la taille d'une tête d’épingle qui vont confluer en plaques érythémateuses ou érythémato-squameuses psoriasiformes hérissés de papules folliculaires bien visibles en périphérie. À cette éruption cutanée est associée une kératodermie palmo-plantaire lisse, particulière par sa couleur jaune orangée. Le cuir chevelu est le siège d'une importante desquamation [1]. L'aspect histologique est souvent non spécifique. La papule cornée folliculaire est constituée d'une importante hyperkératose s'enfonçant dans l'infundibulum pilaire et engainant le poil qui peut être atrophié. L’épiderme adjacent est le siège d'une acanthose modérée et d'une hyperkératose comportant souvent des foyers de parakératose. Dans le derme un infiltrat lymphohistiocytaire modéré, périfolliculaire et périvasculaire peut être observé. L’étiopathogénie de cette affection qui est considérée comme idiopathique reste inconnue, cependant l'association PRP et autres pathologies a été déjà décrite notamment les infections comme les hépatites [2] et le HIV [3], les néoplasies [4], les arthrites inflammatoires et les pathologies auto-immunes telles que la dermatomyosite [5], le vitiligo [6], le lupus subaigu [7], l'hypothyroïdie, la maladie cæliaque et le diabète [8]. L'association PRP et myasthénie comme dans notre observation est rare. D'après notre connaissance un seul cas de PRP myasthénie grave a été rapporté dans la littérature avec une bonne évolution après traitement par vitamine A [9]. Ces associations sont en faveur d'une réponse immunitaire anormale vis-à-vis des antigènes et des agents microbiens avec diminution de l'activité des lymphocytes T suppresseurs et augmentation de celle des lymphocytes T helper [6]. La bonne réponse de PRP à l'Azathioprine et au Calcipotriol qui inhibent l'activation des lymphocytes T conforte cette hypothèse [3] et récemment certains cas de PRP ont été traités par les anticorps anti TNF alpha avec bonne évolution suggérant son rôle important dans la physiopathologie de PRP [10].

## Conclusion

La coexistence de PRP et maladies auto-immunes soulève l'hypothèse d'une origine auto-immune de cette pathologie. Cependant vu la rareté de cette pathologie il reste difficile d’établir avec précision l'explication de cette association.
